# Putting the “mental” back in “mental disorders”: a perspective from research on fear and anxiety

**DOI:** 10.1038/s41380-021-01395-5

**Published:** 2022-01-26

**Authors:** Vincent Taschereau-Dumouchel, Matthias Michel, Hakwan Lau, Stefan G. Hofmann, Joseph E. LeDoux

**Affiliations:** 1 Department of Psychiatry and Addictology, Université de Montréal, Montreal, Canada; 2 Centre de Recherche de l’Institut Universitaire en Santé Mentale de Montréal, Montreal, Canada; 3 Department of Philosophy, New York University, New York, NY 1003 USA; 4 RIKEN Center for Brain Science, Wako, Japan; 5 Department of Clinical Psychology, Philipps-University Marburg, Marburg, Germany; 6 Department of Psychological and Brain Sciences, Boston University, Boston, MA USA; 7 Center for Neural Science and Department of Psychology, New York University, New York, NY 1003 USA; 8 Department of Psychiatry, and Department of Child and Adolescent Psychiatry, New York University Langone Medical School, New York, NY 1003 USA

**Keywords:** Psychology, Neuroscience

## Abstract

Mental health problems often involve clusters of symptoms that include subjective (conscious) experiences as well as behavioral and/or physiological responses. Because the bodily responses are readily measured objectively, these have come to be emphasized when developing treatments and assessing their effectiveness. On the other hand, the subjective experience of the patient reported during a clinical interview is often viewed as a weak correlate of psychopathology. To the extent that subjective symptoms are related to the underlying problem, it is often assumed that they will be taken care of if the more objective behavioral and physiological symptoms are properly treated. Decades of research on anxiety disorders, however, show that behavioral and physiological symptoms do not correlate as strongly with subjective experiences as is typically assumed. Further, the treatments developed using more objective symptoms as a marker of psychopathology have mostly been disappointing in effectiveness. Given that “mental” disorders are named for, and defined by, their subjective mental qualities, it is perhaps not surprising, in retrospect, that treatments that have sidelined mental qualities have not been especially effective. These negative attitudes about subjective experience took root in psychiatry and allied fields decades ago when there were few avenues for scientifically studying subjective experience. Today, however, cognitive neuroscience research on consciousness is thriving, and offers a viable and novel scientific approach that could help achieve a deeper understanding of mental disorders and their treatment.

## Introduction

Problems related to fear and anxiety are among the most prevalent forms of mental illnesses [1] and have been the subject of much research in animals [2–8] and humans [9, 10]. The success of this pre-clinical research has substantially influenced modern clinical interventions [11–19]. Yet, treatments remain less satisfactory than patients and therapists would like [20–24]. We propose here that one factor, more than all others, has contributed to this state of affairs: the systematic marginalization of the subjective experience of patients as a research topic and treatment target.

Modern theories of emotion started in the late nineteenth century with Charles Darwin [25] and William James [26]. Both emphasized subjective experience but in different ways. For Darwin the mental state of emotion caused behavioral and physiological responses in the body, while for James the body responses defined the mental state. Contemporary theories of human emotions, including fear and anxiety, still emphasize the relation between subjective experience, overt behavior, and physiological changes [26, 27]. But the subjective component, typically assessed via verbal report, has been viewed as no more important than the others, and, in fact, has often been least valued by scientists. This bias has its roots in the early twentieth century when behaviorists, because of free-wheeling attribution of mental states as causes of human and animal behavior [28, 29], shunned subjective experience as a scientific construct [30]. The trend continued in the middle of the century, when physiological psychologists, mostly from behaviorist backgrounds, began studying brain mechanisms of overt behavior in animals using the methods of behaviorism and embracing its disdain for anything mental [31–34]. Although cognitive science was emerging as a new approach to the mind around this time, it treated the mind as a system that processes information rather than one that generates subjective experiences [35].

Throughout much of the first half of the twentieth century, the subjective mind was nevertheless alive and well in psychiatry, which was dominated by the psychoanalytic approach initiated by Sigmund Freud. But clinical psychologists in the 1950s and 60s began designing new therapies based on behavioral principles [36, 37]. And biologically oriented psychiatrists were searching for medicinal treatments, often through behaviorist-inspired studies of animals [38–40]. Proponents of these approaches were motivated, in part, by a desire to distance themselves from Freud’s legacy. While they had cause to desire a fresh start, rather than simply distancing themselves from Freud’s view of the mind, they dismissed the central role of subjective mental states in mental illness.

During this same time, the cognitive approach to therapy was also emerging in the hands of Albert Ellis [41] and Aaron Beck [42], both of whom were initially trained as psychoanalysts. Their twist was to change the focus of subjective distress from unresolved unconscious conflict to maladaptive beliefs and automatic thoughts. However, over the subsequent years, the popularity of the medical model of psychiatry came to be the standard of how to evaluate therapeutic outcomes, and even cognitive approaches began to treat subjective experience as just another factor that contributed to the “disease”. As a result, the tendency to marginalize subjective experience is the norm rather than the exception in the field, despite the fact that the way a patient feels subjectively is a major factor that leads them to seek help, and also shapes their evaluation of whether the treatment has been effective.

Clinicians, of course, have always wanted their patients to feel better as a result of their therapies. But because of the inconsistencies they observed in the self-report of patients during clinical interviews, self-report acquired a bad reputation. As we will see, this was supported by research that questioned the reliability of self-report. But in throwing the baby out with the bathwater, important, empirically useful aspects of self-report were ignored. As therapy became more evidence-based, and insurers demanded objective treatment targets to evaluate treatment success, the scientific merit of self-reports was further marginalized (as reflected in the NIMH RDoC initiative [43, 44]).

In this paper, we propose that the marginalization of subjective experience in modern psychology, neuroscience, and psychiatry made it inevitable that the treatments developed and implemented would be less effective than desirable. Specifically, we suggest that treatments designed to target easily measurable behavioral and physiological manifestations, while useful for treating behavioral and physiological symptoms, are problematic as an approach to improving subjective well-being.

We will use fear to make our case, and will argue that, contrary to long-standing and current trends, subjective fear is *not* just another factor in the emotion fear; it is what the emotion fear is [3, 22, 45]. We believe that the acceptance of this view would allow a deeper understanding of the relation of adaptive to pathological fear and anxiety, and pave the way for new, more effective, approaches for the treatment of prevalent and troubling conditions involving these mental states.

Before laying out our arguments, it is important to point out that fear and anxiety, though related, are different states (see [3]). Nevertheless, because these terms have often been used interchangeably in the historical literature, we use the terms interchangeably when referring to historical points.

## The disease model of fear and anxiety

Early nosological systems emphasized deep-seated psychodynamic conflicts as the latent causes of dysfunction in multiple mental illnesses. Today, the American Psychiatric Association [46, p. 20] defines mental disorders, including anxiety disorders, as “a syndrome characterized by clinically significant disturbance in an individual’s cognition, emotion regulation, or behavior that reflects a dysfunction in the psychological, biological, or developmental processes underlying mental functioning”. Contemporary classification systems, such as the International Classification of Disease (ICD-11) and the Diagnostic and Statistical Manual of Mental Disorders (DSM-5), explain “dysfunction” by adopting a medical illness model that assumes that symptoms reflect latent disease entities. In this perspective, anxiety disorders are a consequence of abnormal brain circuits, neurotransmitters, genes, and/or other biological abnormalities [43]. It is assumed that pharmacological and/or psychological interventions can be effective treatments because they correct such pathophysiological conditions.

This medical perspective gave rise to the commonly used approach of evaluating the involvement of pharmaceutical and other biological targets using behavioral tests in animals before conducting clinical trials in humans. It was assumed that interventions that proved effective and safe in pre-clinical studies could then be tested in human patients. Because animals lack the ability to give verbal self-reports of their inner feelings, behavioral and physiological responses could be used as proxies for subjective experience.

But contrary to the predictions of the medical model, decades of research have failed to discover new, efficacious pharmacological treatments [20, 21, 47–49]. As a result, the pharmaceutical industry has been eliminating or reducing efforts in psychiatric drug discovery [23, 24, 50]. According to Steven Hyman [51], former director of the National Institute of Mental Health, the failure of the pharmaceutical industry in the area of psychiatric research is leading to a global healthcare crisis since psychiatric illness is the world’s leading cause of disability, and is resulting in enormous societal burden.

Why has this effort failed? We believe that it was, in fact, doomed from the start by its commitment to a simplistic view of human suffering [52]. Specifically, the medical model of fear depends too heavily on the assumption that all three aspects of fear (subjective, behavioral, physiological) have a common origin—a fear circuit—in the brain. For instance, the DSM-5 describes fear as including “surges of autonomic arousal necessary for fight or flight, thoughts of immediate danger, and escape behaviors” ([46], p. 189). This view posits that all three aspects are manifestations of the same underlying circuits. Since we humans are assumed to have inherited our “fear circuits” from our mammalian ancestors, interventions that are effective at normalizing behavioral and physiological proxies in rats and mice should be effective in treating fear and anxiety disorders. To the extent that subjective feelings are also troubling, treating the fear circuit should address those, since fear, like behavioral and physiological responses, is a product of the fear circuit. As noted above, we do not share this view and suggest that subjective and objective responses be addressed separately.

### Terminological confusion in the study of “fear”

Fear has received more scientific attention than any other emotion. But there have been two conflicting approaches. The first started with Darwin, who defined emotions like fear as “states of mind” that we have inherited from our mammalian ancestors by virtue of having inherited some feature of their nervous system [25]. This meshed well with the emphasis on consciousness by both animal and human psychologists in the late nineteenth century [53]. The second approach began in the early twentieth century when the “behaviorists“ called out psychologists for their rampant and often unjustified use of consciousness as explanation of behavior.

The behaviorists dominated psychology for the next several decades. Consequently, the vast majority of researchers in animal psychology from the 1920s into the 1960s, and even into the 1970s, were either behaviorists, or trained by behaviorists. Despite their disdain for the use of subjective states to explain behavior, behaviorists nevertheless retained the use of subjective state terms (e.g., fear, hunger) to describe the motivations underlying behavior. These researchers did not typically mean that a subjective state of fear or hunger was responsible for avoidance of danger or approach to food [54, 55]. Instead, these terms were said to refer to hypothetical intervening variables that connected stimuli with responses [56]. For example, fear was a functional relation between a dangerous or threatening stimulus and a protective (defensive) response [57–59].

Meanwhile, biologists studying behavior worked more in the tradition of Darwin. One group, the ethologists, opposed the behaviorist lack of concern with species differences in behavior, but tended to side with them regarding subjective experience [60]. Another group, physiologists, studied the brain mechanisms of emotional behavior. The well-known work of Cannon, Bard, Hess, Kluver, and Bucy revealed the role of the hypothalamus and temporal lobe in aggressive and defensive behaviors (see [2]). These researchers were unconstrained by behaviorism and some freely treated the emotional behaviors they studied as indicators of subjective feelings of rage or fear.

In the 1950s some behaviorists became physiological psychologists. That is, their intervening variables became physiological states in brain areas. This move was inspired by the work of the physiologists mentioned above. But physiological psychologists mostly remained true to their behaviorist legacy, treating the physiological factors they studied as non-subjective motivational states, at least initially.

The leading behavioral approach for studying “fear” in animal psychology from the 1940s through the 1970s was Mowrer’s [61–64] avoidance procedure ([65, 66] For a review, see [67]). Mowrer proposed that rats are motivated to avoid aversive stimuli (electric shocks) by “fear.” Behaviors that led to successful escape from, and later avoidance of, the aversive stimulus were reinforced by “fear” reduction. An important finding was that early in training heart rate rises, but then once the avoidance response is well-established the rate normalizes [65, 66, 68]. This was interpreted to mean that “fear” leads to the elevation of heart rate. Successful avoidance is then accompanied by a reduction of “fear,” and a decrease in heart rate follows [69].

Behaviorists like Mowrer treated fear as an intervening variable [57, 64]. What did this mean? The natural assumption among behaviorists at the time was that fear was a non-subjective state that controls behavior. But as behaviorism became a less dominant force in psychology, even some behaviorists began to speak about “fear” as if they meant subjective fear, using expressions like “frightened rats” or “rats frozen in fear” [65, 70]. Often within a single paper “fear” seemed to refer to a non-subjective state in some sentences, while in others it seemed to imply that the animals were subjectively afraid. This was likely as much about ideology as about how difficult it is to refrain from reverting to the use of an everyday vernacular term for a mental state in a non-mental state way.

Some two decades after starting the field, Mowrer clarified his position, noting that rats freeze and avoid “by-cause of” fear; for him, in other words, “fear” always meant conscious fear [71]. Though one could have read this between the lines that he penned over the years, the field seems to have been blinded to what he was really saying by their ideology.

Mowrer’s work not only impacted research on animal behavior but also came to be the way that fear was viewed by clinicians. From the beginning, Mowrer was interested in avoidance learning in animals as a tool for understanding pathological human anxiety [63]. At that time, Freud’s psychoanalytic approach was the dominant clinical approach, and Mowrer proposed that principles of behavioral learning could improve clinical treatments [72]. Subsequently, Mowrer’s colleague, Neal Miller, continued this effort, writing a book called *Personality and Psychotherapy* with the psychoanalyst John Dollard [73]. But by then psychoanalysis was on the wane, and these efforts, rather than broadening the scope of psychoanalytic treatment, paved the way for the emergence of behavior therapy [36], and then cognitive-behavioral therapy [41, 74]. Mowrer’s two-factor theory continues to be cited in contemporary clinical understanding of anxiety [75–77].

The terminology of fear became even more confusing in the 1970s with the revival of the Darwinian approach adopted by psychological researchers in guise of basic emotions theory [78–81]. Fear, in this perspective, was an innate emotion inherited from mammalian ancestors in the form of a neural “affect program“ or “emotion operating system.” Jaak Panksepp [80, 81], for example, used evidence implicating the amygdala and periaqueductal gray regions of the brain in the defensive behaviors of rats as the basis for postulating that homologous emotion operating systems underlies, not just behavioral and physiological responses, but also the subjective experience of fear in rats and humans alike.

Many working on the circuits underlying defensive behavior in the behaviorist-oriented physiological psychology tradition at this time did not bother to address the issue of what fear meant, since conscious fear was a non-starter, and they just assumed it was a non-subjective physiological amygdala state. Nevertheless, when discussing the implications of behavioral studies in animals for understanding fear and anxiety as clinical problems, they often talked about fear in the colloquial way.

Because the colloquial way is the way most people, including lay people, journalists, and most scientists not in the fear field, think about fear, the public conversation about fear circuits was about conscious fear. The result was that the idea of the amygdala as the seat of fearful feelings in the brain became a cultural meme, one that also implied that drugs or other treatments that target the amygdala could make people less fearful and anxious [18, 82, 83].

### Lang’s three-systems model of fear

As a result of the inconsistent use of the term “fear” in the 40s and 50s, some researchers in the 1960s began to wrestle anew with fear as a scientific construct. The work of Peter Lang was particularly important.

Lang noted a number of instances in the literature which showed that subjective fear experiences (as measured by verbal reports) did not correlate well with objective and measurable behavioral responses (e.g., avoidance behavior) and physiological changes (e.g., in heart rate) [84–86]. Accordingly, he was critical of the importance that some clinicians placed on subjective states over behavior and physiology.

Under the lingering influence of behaviorism and the growing influence of the new cognitive movement in psychology, Lang proposed that verbal behavior should be repurposed. Rather than using it as a way to assess intangible subjective experiences, it should be used to track more tangible cognitive processes. Treatment could then be focused on altering verbal behavior, which would, in turn, reflect changes in the underlying cognitive processes, much like the way that treatments that change overt behavior or physiological arousal do so because they change underlying processes.

Expressing his scientific distaste for subjective experience, Lang noted: “whether seen as causes or consequences, feelings are beyond the pale of direct scientific inquiry” ([87], 124). Fear, he said, “is not some hard phenomenal lump that lives inside people” [87]. Instead, it is a response expressed in three response systems: verbal (cognitive), overt motor, and somatic. The responses corresponding with these were self-report for the cognitive system, behavior (especially avoidance behavior) for the overt motor system, and physiological changes for the somatic system. Therapy, he argued, should focus on changing the specific response systems, since each contributes separately to the overall intensity of fear.

### Discordance and desynchrony

Lang’s, “three-system model” stimulated much clinical research and theorizing [88–94]. While his views had their greatest impact on clinical research, they also affected basic research in psychology and neuroscience.

One problem was that Lang’s terminology (cognitive, overt motor, and somatic responses) was a bit unclear. For example, “somatic” is more typically used to refer to skeletal-motor responses underlying overt behavior than to visceral autonomic responses (e.g. [95]). As we proceed we will, therefore, use a more straightforward set of terms: self-report, behavioral, and physiological responses. By “self-report” we specifically mean verbal reports resulting from conscious fear experiences. Such reports can be interpreted as indicating that the person is having, or has had, a subjective experience of fear in the presence of a threatening stimulus or situation. By “physiological responses” we mean increases in skin conductance, heart rate or other visceral changes in the body in response to threatening stimuli or situations. By “behavioral responses” we mean threat-elicited reactions (freezing, flight), as well as threat-motivated instrumental behaviors (avoidance), expressed in the presence of threatening stimuli or situations [96].

We will use this terminology to discuss two kinds of discrepancies in this literature. The lack of concordance between the three measures of “fear” at a given time is referred to as *discordance* [90]. There are many examples of discordance in the literature [97]. For instance, in the presence of threat, patients have reported high levels of subjective fear, and yet demonstrate normal or even low levels of physiological threat responses (e.g. heart rate or skin conductance measures), while others show the opposite pattern [89, 98–102]. Other forms of discordance have been observed following pharmacological interventions [103]. Medications, such as beta-blockers, for example, can dampen the hyperreactivity of the autonomic nervous system (e.g. heart rate acceleration) or behavior (e.g., trembling hands, fidgeting) in the presence of actual or perceived threats without necessarily affecting the subjective experience of anxiety [104].

Discordance is distinguished from the phenomenon of desynchrony. The latter refers to variations in the levels of the three measures over time. For example, a patient undergoing behavioral therapy for exaggerated fear or anxiety may first show signs of reduced behavioral and physiological symptoms, and gradually demonstrate changes in self-reports of fear later on. This was reported by Lang in early clinical trials [105]. Another example pertains to the desynchrony between avoidance behaviors and subjective fear. For instance, the presentation of aversive stimuli often generates both avoidance behaviors and subjective reports of fear. But, successful avoidance will typically lead to a decrease in fear reports while avoidance behaviors can persist over extended periods of time [106].

Cases of discordance and desynchrony emphasize that behavioral and physiological responses that are sometimes correlated with subjective fear should *not* necessarily be interpreted as indicating that the person is consciously experiencing subjective feelings of fear per se [54, 55, 107]. In fact, for the sake of clarity, if nothing else, we maintain that the mental state term “fear” should be reserved for the mental state, and behavioral and physiological responses should be referred to as “threat” or “defense“ responses.

Considerable confusion in the discordance and desynchrony literature has also resulted from a failure to recognize that in threatening situations a variety of behaviors can result [96]. Species-typical (innate) reactions (e.g. freezing behavior) are automatically elicited by unlearned or conditioned stimuli, while instrumental responses (e.g. avoidance) are acquired by their consequences and are emitted in appropriate situations. Species-typical reactions to unlearned or conditioned stimuli have reliable physiological correlates that are “wired in” as part of the “defense reaction” [108], but most avoidance and other instrumental responses do not, since these can be achieved in many ways [109]. This may account for the poor correlation often observed between physiological measures and avoidance behavior [106]. Furthermore, instrumental avoidance responses, though often treated as a single class of response, can be due to habit learning, goal-directed action learning, or cognitive deliberation, each of which involves different neural circuits [96, 110]. Future studies should adopt a more subtle approach to behavioral measures.

### Conceptual challenges

Given that discordance and desynchrony between responses occur, the key question is whether self-report, behavior, and physiology should be interpreted as indicating the existence of different psychological constructs, or whether they should be interpreted as indications of a single multi-faceted underlying construct. This is an issue of construct validity [111].

Construct validation is typically achieved by establishing robust correlations between the results of different tests purporting to measure the same construct. If measures of self-report, physiological activity, and behavioral responses were systematically correlated, it would be relatively straightforward to interpret them as collectively reflecting a single underlying construct. However, studies have typically found that self-report shares only a modest part of its variance with other measures [112], with the most optimistic estimates indicating about 27–28% of shared variance [113].

There are two main views regarding the interpretation of discordance and desynchrony in this literature (for an in-depth discussion of these in relation to construct validity in “fear” studies, see [114]). The first attempts to salvage a singular fear construct, despite the existence of discordance and desynchrony, by arguing that self-report, behavior, and physiology are each indicators of the same underlying construct (fear), but that they differ in the degree of accuracy with which they reflect the construct. The second posits that the three factors are independent, but interacting, constructs.

Those who favor the first view maintain that using self-reports to assess fear in effect amounts to using an inaccurate measurement procedure. For instance, Fanselow and Pennington (2018) [82, p. 27] argue that the amygdala is a “fear generator” that controls all three response types, but that the most reliable measures are the behavioral and physiological outputs. They write that “the additional machinery needed to generate subjective report probably adds additional noise, rendering it… a less pure and objective measure of fear.” In this view, cases of discordance and desynchrony are explained away as being due to the fact that self-reports are the least accurate of the three measures of fear [58, 82, 115]. According to Fanselow and Pennington (2018), emphasizing the subjective experience of fear will “push us back well over a century to what was truly the dark ages of psychiatry” (p. 28).

**Fig. 1:**
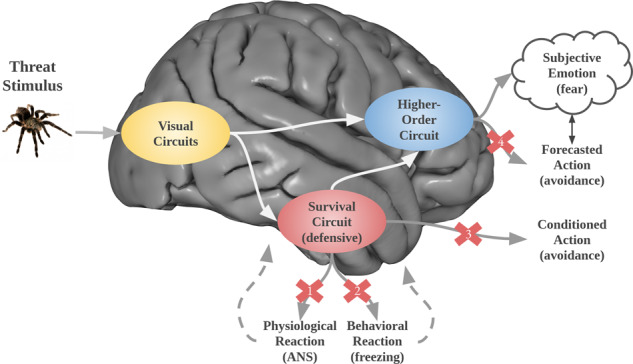
Discordance and desynchrony in light of a higher-order perspective. Threatening stimuli often lead to subjective fear via the higher-order circuit, and trigger bodily reactions (behavioral and physiological responses) via the defensive survival circuit, in parallel. This higher-order model can account for situations where subjective and objective responses are discordant or desynchronous. For instance, blocking physiological reactions (X1) dissociates them from conditioned or forecasted actions and/or conscious experiences, while blocking physiological reactions (X2) dissociates those from behavior reactions and/or conscious experience. Similar logic applies to X3 and X4. ANS autonomic nervous system.

In contrast, those who favor the second view posit that cases of discordance and desynchrony indicate the existence of separate factors. For example, LeDoux and colleagues [21, 116–118] argue that while behavioral and physiological responses elicited by threats are products of the amygdala, subjective fear reflects a cognitive interpretation that one is in a situation of potential or actual psychological or physical harm. Such an approach is hardly a fringe idea, as cognitive theories are leading explanations of emotions [119–121]. Recently, the higher-order theory (HOT) of consciousness (see Box 1; [122]), which is usually discussed in relation to visual perception, has been extended as a novel cognitive account of fear and other emotions [116, 123, 124]. According to HOT, consciousness arises when higher-order cognitive structures monitor or meta-represent lower-order information (see Fig. 1). A simple version of the higher-order account would be that signals resulting from the consequences of the behavioral and physiological responses generated by the amygdala in the brain and body are re-represented and contribute to the experience of fear. But the model also includes emotion schema and self-schema, as well as meta-representations of semantic and episodic memories. These representations result in a mental model of the dangerous situation, which can fully account for the subjective experience of fear, even in situations where the amygdala activity and body feedback are absent. That this is necessary is clearly indicated by discordance and desynchrony between subjective fear and body arousal. Antonio Damasio [125] similarly noticed this and proposed “as if body loops” that simulate brain and body activity when these are absent.

The controversy surrounding the two perspectives is in part fueled by the long and complex history of subjective reports [29, 126]. For instance, some social psychologists have suggested that self-reports about the causes of our own actions are often mistaken [127, 128]. The use of self-reports has also been criticized in other disciplines, such as sociology [129], thus indicating that humans sometimes exhibit surprisingly poor capacities for self-knowledge (for a review, see [130]). This evidence could be interpreted as suggesting that subjective reports are systematically inaccurate, and are, therefore, unreliable scientific tools.

However, alleged cases of unreliability are not cases in which subjects report about ongoing conscious experiences, but instead are typically cases in which participants report about the causes of their behaviors [128], or about long-standing psychological attitudes such as their beliefs [130]. Aside from pathological cases (e.g. Anton’s syndrome), or malicious deceit, there is no significant body of empirical evidence to support a general dismissal of subjective reports about conscious experiences such as perceptual experiences, fear or anxiety [131, 132]. As a matter of fact, a wide variety of experiments in fields, such as perceptual psychology [133], and even more germane, the scientific study of emotions [134], rely on experimentally controlled subjective reports about what the subject experiences.

According to Borsboom et al.’s [135, p. 1061] definition of validity, “a test is valid for measuring an attribute if and only if (a) the attribute exists and (b) variations in the attribute causally produce variations in the outcomes of the measurement procedure”. Given that self-reports can be interpreted as resulting from variations in metacognitions (cognitive re-representations) that are directly antecedent to the experience of fear, it follows that self-reports are valid indicators of fear experience. On the other hand, since behavior and physiology can sometimes dissociate from the feeling of fear, interpreting them as reliable indicators of fear, if we follow Borsboom, is invalid, though not necessarily useless.

These observations are in line with the second interpretation of discordance and desynchrony in fear research discussed above. As such, we hold that behavior and physiology, on the one hand, result from threat detection and the activity of defense mechanisms, while self-report, on the other hand, results from the metacognitions upon which subjective experience is based. It follows that self-report, which reflects these metacognitions as well (Fig. 1), is the only valid indicator of fear as a subjective experience.

Box 1 First-order theories vs higher-order theories In consciousness science, one core topic of disagreement pertains to the origin of consciousness in the brain. Here, when we say consciousness, we refer to what is sometimes called phenomenal consciousness, that is the qualitative or phenomenal “feel” of experiences. For example, looking at a sunset has a subjective character that can be described in terms of “what it feels like”. This is different from what can be called states of consciousness which are studied, for instance, in minimally conscious patients or sleep. While undoubtedly important, especially for clinical practices, the study of states of consciousness does not directly address the question of how the brain generates this subjective “feel” of things.

While many theories of phenomenal consciousness make vastly different predictions from one another [156], they can be broadly divided in two categories. First-order theories, such as recurrent processing theory [157–160], posit that consciousness originates in brain regions specialized in the processing of a given type of information (for instance, visual or auditory cortices). As we saw in the main text, some authors have suggested that the amygdala might be such a first-order structure in the subjective experience of fear [81, 82, 161]. Another first-order theory, the global neuronal workspace theory, posits that the activity within first-order structures becomes conscious when it is made available to other brain regions through a global broadcasting mechanism.

On the contrary, higher-order theories suggest that these first-order structures may not be sufficient for the information to become conscious [122, 162, 163]. They posit that some additional cognitive processes in other regions may be needed in order to monitor the information. In this perspective, subjective experience arises from a mechanism closely related to metacognition, which also involves the monitoring of one’s own cognitive and sensory processing [164]. As such, the information represented in first-order structures should remain unconscious if no higher-order processing is involved. With respect to fear, this view posits that the amygdala non-consciously controls defensive behavioral and physiological responses to threats, but that higher-order processes are required in order to generate the subjective experience to the same threatening stimulus [54, 55, 107, 116, 163]. In this view, the re-representation of the first-order information (often termed as meta-representation) is a non-conscious antecedent to consciousness. We suggest that treatment strategies that target both the subjective (conscious experience) and objective (behavioral and physiological responses) will be more effective than approaches that primarily focus on objective responses. We also suggest that measures of discordance and desynchrony can provide additional indicators of treatment progress.

### Clinical pragmatism

In addition to its scientific merits, our view of the subjective fear construct is consistent with the way patients express their concerns in clinical settings, and is often what they care most about. From a clinical perspective, a problem usually only reaches the level of clinical significance if it is associated with significant subjective distress and/or interferes with the person’s life. Without the subjective experience of distress, it is very difficult to conclude that an individual suffers from an emotional disorder. This is why subjective distress is a core feature of the definition of an emotional disorder (e.g., in the DSM-5). From this perspective, self-report is the most direct measure of the patient’s problem and treatment efficacy. Thus, whether implicit or explicit, the subjective experience of the patient has been the focal point of all mental disorders, especially emotional disorders.

At the same time, self-report data rarely determine the clinical status directly. Instead, the patient’s subjective report is filtered and interpreted by a clinician to derive a clinical assessment. This is in part because clinicians have long recognized that relying only on self-report in their clinical assessment presents some limitations. As we discussed in the “Conceptual challenges” section, self-report about recalled causes of past behaviors [128] or about beliefs [130] can sometimes be misleading. Such observations, together with the influence of the behaviorist movement, fueled a general trend in psychiatry research to look for objective (behavioral and physiological) markers of pathology. For instance, although the Research Domain Criteria initiative (RDoC) of the NIMH (the National Institute of Mental Health in the United States) [43, 44] purports to recognize the importance of human psychology, its view of self-report is ambivalent at best: “experiential claims represent a kind of “folk” psychology of the self that should [not be] assumed veridical.” It also acknowledges that these claims should not be “simply discounted” [44, p. 292]. As such, the ultimate goal in psychiatry research in modern times has chiefly been to identify biological markers of mental disorders, akin to other medical diseases.

Some have resisted this trend and advocated for the importance of subjective reports [136–138]. For instance, Edna Foa [139], a leading clinical researcher, noted that self-report generates “valid measures of key constructs, some of which cannot be measured in any other way, and sometimes are the best measure of the construct of interest”. Similarly, we [3, 21, 55] and others [45] argued that emotions are first and foremost subjective experiences. As a result, self-report should play a significantly more prominent role in clinical practice. It can be collected through clinical interviews, daily diaries, in vivo exposure, computerized tasks, or using virtual reality approaches. And given that we now have better understanding of the various factors that affect the validity and reliability of self-report, we can work toward improving clinical tools, paving the way for more rigorous and valid assessments of subjective experience in clinical practice.

By distinguishing between physiological, behavioral, and self-report measures, fear and anxiety research can use valid and reliable procedures for addressing each of those constructs when needed. For instance, unlike physiological responses, subjective ratings during an extinction procedure (i.e., expectancy of the unconditioned stimulus) are predictive of post-exposure affective ratings, a clinically meaningful measure associated with the relapse of fear [140]. Importantly, this association was observed even though subjective ratings were also correlated with physiological responses at various stages of the experiment. Furthermore, another line of evidence comes from a recent meta-analysis indicating that psychotherapy and pharmacotherapy may have very different effects in the brain [141]. More precisely, the results suggest that psychotherapy might target cognitive processes and schema in the prefrontal cortex while antidepressant medication might primarily affect the amygdala and basal ganglia. As we saw above, there are reasons to believe that objective measures may primarily originate from the defensive survival circuit that includes the amygdala while the subjective experience is likely generated by the higher-order circuit that includes the prefrontal cortex [141–143]. As such, these examples highlight the added values of considering the three constructs separately as they each provide distinct information and may require different treatment strategies.

Furthermore, by studying how discordance and desynchrony between the three measures naturally occur it may be possible to tailor therapies to the individual needs of patients [21, 144–146]. This idea was notably put forward some time ago by Rachman [89] and Michelson [100] who suggested that behavioral therapy may be particularly effective if a patient has exaggerated behavioral or physiological responses, but low levels of self-reported fear. Such “tailored” approach must however be used with caution as treating exclusively the objective response systems may lead the subjective system to relapse, and vice versa [21].

Early reports also revealed gender differences in discordance and desynchrony [147]. In some situations, men showed lower self-reported levels of negative emotions compared to women even when their physiological responses were high [148]. As one can imagine, effects like these may well be modulated by age and cultural factors. If we can track what the systematic factors modulating the effects on discordance are, this may help establish that discordance is a real, meaningful phenomenon, and not just due to the noisiness of individual measures. Additionally, such findings may help achieve a better understanding of underlying mechanisms.

Once individuals are identified as having higher degrees of discordance and/or desynchrony, it would be possible to examine how their brain’s structure and physiology might be associated with such variations. In Taschereau-Dumouchel et al. (2020) [142] we identified brain regions that are specifically important for decoding self-report vs physiological responses to threat. Studying the connectivity between these different regions—as assessed by structural imaging based on diffusion, or resting-state fMRI data—may also predict individual differences in discordance and desynchrony [145, 146]. Similarly, machine-learning algorithms trained to predict self-report, physiology and behavior [142, 149] could also help us reveal the brain mechanisms associated with discordance and desynchrony. Studying such individual differences in brain processes might therefore help us better understand how discordance and desynchrony are associated with pathological conditions.

As such, distinguishing between the three measures might have great clinical benefits. At the same time, we should not lose sight of the fact that they are related constructs, in part because they are consequences of the same external stimulus. And although the brain processes underlying each are separate, they interact.

### Moving forward

To this day, the role of subjective experience in leading theories of emotions remains marginalized. Basic emotions theorists have tended to emphasize the facial expression of emotions, and to a lesser extent, autonomic responses to a greater degree than subjective experiences [79, 150]. Cognitive appraisal theorists give more weight to subjective experience than basic emotions theories, but they typically treat it as one component among several that collectively constitute an emotion [151]. Cognitive construction theories, on the other hand, respect the centrality of subjective experience, and treat it as a conceptualized byproduct of valence and arousal [152]. Our higher-order theory is, in some sense, constructionist and conceptual in nature, but it has a broader view of the non-conscious precursors [107, 116, 118, 123, 145, 153] and it highlights the idea that the conscious experience is the emotion (also see [45]).

Considering the marginalized role of subjective experience in emotion research, and the fact that objective measures of physiology and behavior are relatively poor indicators of subjective suffering, we [3, 21, 55] and others [45], have felt compelled to raise concerns. In some related fields, this issue has long been taken seriously. For instance, in the study of pain, self-report is the traditional gold standard in part due to well-known cases of discordance (see [154]), not unlike those we emphasized. But research on many mental health disorders has unfortunately not generally benefited from a similar epiphany. With discussions about other disorders also emerging [155], we think that the time is right for a change.

Progress in the scientific study of consciousness, and recent work applying this knowledge to explore emotional consciousness, opens the door for a new beginning for designing treatments that will hopefully better target subjective aspects of mental disorders. To succeed, though, this will require a reassessment of some of the implicit assumptions of the behaviorist and medical model legacies, both of which linger as sources of unconscious inferences that guide research and theory. However, we are confident that the approach we tout will lead to new interventions, including personalized ones, capable of tackling mental health disorders in a more complete fashion.

## References

[1] Kessler RC, Berglund P, Demler O, Jin R, Merikangas KR, Walters EE (2005). Lifetime prevalence and age-of-onset distributions of DSM-IV disorders in the National Comorbidity Survey Replication. Arch Gen Psychiatry.

[2] LeDoux JE. The emotional brain: the mysterious underpinnings of emotional life. Simon and Schuster; 1996.

[3] LeDoux JE. Anxious: using the brain to understand and treat fear and anxiety. Penguin; 2015.

[4] Morrison FG, Ressler KJ (2014). From the neurobiology of extinction to improved clinical treatments. Depress Anxiety.

[5] Perusini JN, Fanselow MS (2015). Neurobehavioral perspectives on the distinction between fear and anxiety. Learn Mem.

[6] File SE (2001). Factors controlling measures of anxiety and responses to novelty in the mouse. Behav Brain Res.

[7] Lang PJ, Davis M (2006). Emotion, motivation, and the brain: reflex foundations in animal and human research. Prog Brain Res.

[8] Gray JA, McNaughton N. The psychology of anxiety and enquiry in to the functions of the septo hippocampus system. New York: Oxford University Press; 2000.

[9] Hartley CA, Phelps EA. Changing fear: the neurocircuitry of emotion regulation. Neuropsychopharmacology. 2010;35:136–46. 10.1038/npp.2009.121 PMC3055445 19710632

[10] Büchel C, Dolan RJ (2000). Classical fear conditioning in functional neuroimaging. Curr Opin Neurobiol.

[11] Grupe DW, Nitschke JB (2013). Uncertainty and anticipation in anxiety: an integrated neurobiological and psychological perspective. Nat Rev Neurosci.

[12] Krystal JH, Deutsch DN, Charney DS (1996). The biological basis of panic disorder. J Clin Psychiatry.

[13] Maren S, Phan KL, Liberzon I (2013). The contextual brain: implications for fear conditioning, extinction and psychopathology. Nat Rev Neurosci.

[14] Craske MG, Kircanski K, Zelikowsky M, Mystkowski J, Chowdhury N, Baker A (2008). Optimizing inhibitory learning during exposure therapy. Behav Res Ther.

[15] Ehlers A, Bisson J, Clark DM, Creamer M, Pilling S, Richards D (2010). Do all psychological treatments really work the same in posttraumatic stress disorder?. Clin Psychol Rev.

[16] Barlow DH, Raffa SD, Cohen EM. Psychosocial treatments for panic disorders, phobias, and generalized anxiety disorder. In: Nathan PE, Gorman JM, editors. A guide to treatments that work, Oxford University Press; 2002.

[17] Hofmann SG, Asmundson GJG, Beck AT (2013). The science of cognitive therapy. Behav Ther.

[18] Milad MR, Quirk GJ (2012). Fear extinction as a model for translational neuroscience: ten years of progress. Annu Rev Psychol.

[19] Mathew SJ, Coplan JD, Gorman JM (2001). Neurobiological mechanisms of social anxiety disorder. Am J Psychiatry.

[20] Griebel G, Holmes A (2013). 50 years of hurdles and hope in anxiolytic drug discovery. Nat Rev Drug Discov.

[21] LeDoux JE, Pine DS (2016). Using neuroscience to help understand fear and anxiety: a two-system framework. Am J Psychiatry.

[22] LeDoux JE, Hofmann SG (2018). The subjective experience of emotion: a fearful view. Curr Opin Behav Sci.

[23] Hyman SE (2012). Revolution stalled. Sci Transl Med.

[24] Miller G (2010). Is pharma running out of brainy ideas?. Science.

[25] Darwin C. The expression of the emotions in man and animals. London: John Murray; 1872.

[26] James W (1884). What is an emotion?. Mind.

[27] Cannon WB (1929). Organization for physiological homeostasis. Physiol Rev.

[28] Keller FS. The definition of psychology. 2nd ed. Appleton-Century-Crofts; 1973.

[29] Boring EG (1953). A history of introspection. Psychol Bull.

[30] Watson JB (1913). Psychology as the behaviorist views it. Psychol Rev.

[31] Stellar E (1954). The physiology of motivation. Psychol Rev.

[32] Olds J (1956). Pleasure centers in the brain. Sci Am.

[33] Weiskrantz L (1956). Behavioral changes associated with ablation of the amygdaloid complex in monkeys. J Comp Physiol Psychol.

[34] Goddard GV (1964). Functions of the amygdala. Psychol Bull.

[35] Miller GA, Galanter E, Pribram KH. Plans and the structure of behavior. Martino Fine Books; 2013.

[36] Wolpe J. Psychotherapy by reciprocal inhibition. Stanford, CA: Stanford University Press; 1958.

[37] Bandura A (1961). Psychotherapy as a learning process. Psychol Bull.

[38] Braslow JT, Marder SR (2019). History of psychopharmacology. Annu Rev Clin Psychol.

[39] Valenstein E. Blaming the brain. New York: Free Press; 1999.

[40] Wittenborn JR. The clinical psychopharmacology of anxiety. Springfield: C.C. Thomas; 1966.

[41] Ellis A (1957). Rational psychotherapy and individual psychology. J Individ Psychol.

[42] Beck AT (1964). Thinking and depression. II. Theory and therapy. Arch Gen Psychiatry.

[43] Insel T, Cuthbert B, Garvey M, Heinssen R, Pine DS, Quinn K (2010). Research domain criteria (RDoC): toward a new classification framework for research on mental disorders. Am J Psychiatry.

[44] Kozak MJ, Cuthbert BN (2016). The NIMH Research Domain Criteria Initiative: background, issues, and pragmatics. Psychophysiology.

[45] Lieberman MD (2019). Boo! The consciousness problem in emotion. Cogn Emot.

[46] American Psychiatric Association. Diagnostic and statistical manual of mental disorders (DSM-5®). American Psychiatric Pub; 2013.

[47] Moncrieff J. The myth of the chemical cure: a critique of psychiatric drug treatment. Springer; 2016.

[48] Harrington A. Mind fixers: psychiatry’s troubled search for the biology of mental illness. W. W. Norton & Company; 2019.

[49] Ivanov I, Schwartz JM. Why psychotropic drugs don’t cure mental illness—but should they? Front Psychiatry. 2021;12. 10.3389/fpsyt.2021.579566 PMC8057300 33889091

[50] Greenberg G. The psychiatric drug crisis. New Yorker. 2013;3.

[51] Hyman SE (2013). Psychiatric drug development: diagnosing a crisis. Cerebrum.

[52] Braslow JT, Brekke JS, Levenson J (2020). Psychiatry’s myopia-reclaiming the social, cultural, and psychological in the psychiatric gaze. JAMA Psychiatry.

[53] Michel M. Consciousness science underdetermined: a short history of endless debates. Ergo. 2019;6.

[54] LeDoux JE (2014). Coming to terms with fear. Proc Natl Acad Sci USA.

[55] LeDoux JE (2017). Semantics, surplus meaning, and the science of fear. Trends Cogn Sci.

[56] Tolman EC. Purposive behavior in animals and man. New York: Century; 1932.

[57] Brown JS, Farber IE (1951). Emotions conceptualized as intervening variables—with suggestions toward a theory of frustration. Psychol Bull.

[58] Kozak MJ, Miller GA (1982). Hypothetical constructs versus intervening variables: a re-appraisal of the three-systems model of anxiety assessment. Behav Assess.

[59] Marx MH (1951). Intervening variable or hypothetical construct?. Psychol Rev.

[60] Tinbergen N. The study of instinct. New York: Oxford University Press; 1951.

[61] Miller NE (1948). Studies of fear as an acquirable drive fear as motivation and fear-reduction as reinforcement in the learning of new responses. J Exp Psychol.

[62] Mowrer OH (1940). Anxiety-reduction and learning. J Exp Psychol.

[63] Mowrer OH (1939). A stimulus-response analysis of anxiety and its role as a reinforcing agent. Psychol Rev.

[64] Mowrer OH, Lamoreaux RR (1946). Fear as an intervening variable in avoidance conditioning. J Comp Psychol.

[65] Rescorla RA, Solomon RL (1967). Two-process learning theory: relationships between Pavlovian conditioning and instrumental learning. Psychol Rev.

[66] Solomon RL, Wynne LC (1954). Traumatic avoidance learning: the principles of anxiety conservation and partial irreversibility. Psychol Rev.

[67] LeDoux JE, Moscarello J, Sears R, Campese V (2017). The birth, death and resurrection of avoidance: a reconceptualization of a troubled paradigm. Mol Psychiatry.

[68] Mineka S (1979). The role of fear in theories of avoidance learning, flooding, and extinction. Psychol Bull.

[69] McAllister WR, McAllister DE. Behavioral measurement of conditioned fear. Aversive conditioning and learning, Elsevier; 1971. p. 105–79.

[70] Bolles RC. Theory of motivation. Harper and Row, New York; 1967.

[71] Mowrer OH. Learning theory and behavior. Wiley; 1960.

[72] Mowrer OH (1948). Learning theory and the neurotic paradox. Am J Orthopsychiatry.

[73] Dollard J, Miller NE. Personality and psychotherapy; an analysis in terms of learning, thinking, and culture. Vol. 488. New York: McGraw-Hill; 1950.

[74] Beck AT (1970). Cognitive therapy: nature and relation to behavior therapy. Behav Ther.

[75] Levis DJ. The case for a return to a two-factor theory of avoidance: the failure of non-fear interpretations. In: Klein SB, Mowrer RR, editors. Contemporary learning theories: pavlovian conditioning and the status of traditional learning theory. Hillsdale: Lawrence Erlbaum Assn.; 1989.

[76] Beckers T, Craske MG (2017). Avoidance and decision making in anxiety: an introduction to the special issue. Behav Res Ther.

[77] Dykman RA, Ackerman PT, Newton JE (1997). Posttraumatic stress disorder: a sensitization reaction. Integr Physiol Behav Sci.

[78] Ekman P. Universals and cultural differences in facial expressions of emotions. In: Cole J, editor. Nebraska symposium on motivation, vol. 19, University of Nebraska Press; 1972. p. 207–83.

[79] Izard CE. The face of emotion New York. New York: Appleton-Century-Crofts; 1971.

[80] Panksepp J (1982). Toward a general psychobiological theory of emotions. Behav Brain Sci.

[81] Panksepp J. Affective neuroscience. Oxford University Press; 1998.

[82] Fanselow MS, Pennington ZT (2018). A return to the psychiatric dark ages with a two-system framework for fear. Behav Res Ther.

[83] Ressler KJ (2020). Translating across circuits and genetics toward progress in fear- and anxiety-related disorders. Am J Psychiatry.

[84] Lang PJ, Lazovik AD, Reynolds DJ (1965). Desensitization, suggestibility, and pseudotherapy. J Abnorm Psychol.

[85] Lacey JI. Psychophysiological approaches to the evaluation of psychotherapeutic process and outcome. In: Rubinstein EA, Parloff MB. editors. Research in psychotherapy. Washington, DC: American Psychological Association; 1959. p. 160–208.

[86] Lang PJ. The mechanics of desensitization and the laboratory study of human fear. In: Franks C, editor. Assessment and status of the behavior therapies, New York: McGraw-Hill; 1969. p. 160–91.

[87] Lang PJ, Miller GA, Levin DN. Anxiety and fear. In: Davidson RJ, Schwartz GE, Shapiro D, editors. Consciousness and self-regulation: Vol. 3: Advances in research and theory. Boston, MA: Springer US; 1983. p. 123–51.

[88] Miller GA, Kozak MJ. Three-systems assessment and the construct of emotion. In: Birbaumer N, Öhman A, editors. The structure of emotion: physiological, cognitive and clinical aspects. Hogrefe & Huber Kirkland, WA; 1993. p. 31–47.

[89] Rachman S (1976). The passing of the two-stage theory of fear and avoidance: fresh possibilities. Behav Res Ther.

[90] Rachman S, Hodgson RI (1974). Synchrony and desynchrony in fear and avoidance. Behav Res Ther.

[91] Borkovec TD. Physiological and cognitive processes in the regulation of anxiety. In: Schwartz GE, Shapiro D, editors. Consciousness and self-regulation: advances in research. Vol. 1. Boston, MA: Springer US; 1976. p. 261–312.

[92] Hugdahl K (1981). The three-systems-model of fear and emotion—a critical examination. Behav Res Ther.

[93] Zinbarg RE (1998). Concordance and synchrony in measures of anxiety and panic reconsidered: a hierarchical model of anxiety and panic. Behav Ther.

[94] Foa EB, Kozak MJ (1986). Emotional processing of fear: exposure to corrective information. Psychol Bull.

[95] Romer AS. The vertebrate as a dual animal—somatic and visceral. In: Dobzhansky T, Hecht MK, Steere WC, editors. Evolutionary biology, Springer; 1972. p. 121–56.

[96] LeDoux JE, Daw ND (2018). Surviving threats: neural circuit and computational implications of a new taxonomy of defensive behaviour. Nat Rev Neurosci.

[97] Hollenstein T, Lanteigne D (2014). Models and methods of emotional concordance. Biol Psychol.

[98] Davidson RJ, Schwartz GE. The psychobiology of relaxation and related states: a multi-process theory. In: Mostoesky DI, editor. Behavior control and modification of physiological activity. Englewood Cliffs, NJ: Prentice-Hall; 1976. p. 399–442.

[99] Lang PJ. Physiological assessment of anxiety and fear. In: Cone JD, Hawkins RP, editors. Behavioral assessment: new directions in clinical psychology. New York: Brunner-Mazel; 1977. p. 178–95.

[100] Michelson L (1984). The role of individual differences, response profiles, and treatment consonance in anxiety disorders. J Behav Assess.

[101] Ost LG, Jerremalm A, Johansson J (1981). Individual response patterns and the effects of different behavioral methods in the treatment of social phobia. Behav Res Ther.

[102] Ost LG, Johansson J, Jerremalm A (1982). Individual response patterns and the effects of different behavioral methods in the treatment of claustrophobia. Behav Res Ther.

[103] Gerrans P, Scherer K (2013). Wired for despair the neurochemistry of emotion and the phenomenology of depression. J Conscious Stud.

[104] Steenen SA, van Wijk AJ, van der Heijden GJMG, van Westrhenen R, de Lange J, de Jongh A (2016). Propranolol for the treatment of anxiety disorders: systematic review and meta-analysis. J Psychopharmacol.

[105] Lang PJ, Lazovik AD (1963). Experimental desensitization of phobia. J Abnorm Soc Psychol.

[106] Gray JA. The psychology of fear and stress. London: Weidenfeld & Nicolson; 1971.

[107] LeDoux JE (2012). Rethinking the emotional brain. Neuron.

[108] Hilton SM, Zbrozyna AW (1963). Amygdaloid region for defence reactions and its efferent pathway to the brain stem. J Physiol.

[109] Cohen DH, Obrist PA (1975). Interactions between behavior and the cardiovascular system. Circ Res.

[110] Balleine BW, Dickinson A (1998). Goal-directed instrumental action: contingency and incentive learning and their cortical substrates. Neuropharmacology.

[111] Cronbach LJ, Meehl PE (1955). Construct validity in psychological tests. Psychol Bull.

[112] Barrett LF (2009). Variety is the spice of life: a psychological construction approach to understanding variability in emotion. Cogn Emot.

[113] Friedman BH, Stephens CL, Thayer JF (2014). Redundancy analysis of autonomic and self-reported, responses to induced emotions. Biol Psychol.

[114] Schaffner KF. A comparison of two neurobiological models of fear and anxiety: a ‘construct validity’ application? Perspect Psychol Sci. 2020;2020. 10.1177/1745691620920860. 10.1177/1745691620920860 32598853

[115] Lonsdorf TB, Merz CJ, Fullana MA (2019). Fear extinction retention: is it what we think it is?. Biol Psychiatry.

[116] LeDoux JE, Brown R (2017). A higher-order theory of emotional consciousness. Proc Natl Acad Sci USA.

[117] LeDoux JE (2021). What emotions might be like in other animals. Curr Biol.

[118] LeDoux JE. Thoughtful feelings. Curr Biol. 2020;30:R619–R623. 10.1016/j.cub.2020.04.012 32516605

[119] Schachter S, Singer JE (1962). Cognitive, social, and physiological determinants of emotional state. Psychol Rev.

[120] Ortony A, Clore GL (1989). Emotions, moods, and conscious awareness; comment on johnson-laird and oatley’s ‘the language of emotions: an analysis of a semantic field’. Cognition Emot.

[121] Barrett LF, Russell JA, editors. The psychological construction of emotions. Guilford Press; 2015.

[122] Rosenthal D. Consciousness and mind. Oxford University Press; 2005.

[123] LeDoux JE (2020). How does the non-conscious become conscious?. Curr Biol.

[124] LeDoux JE, Brown R, Pine D, Hofmann SG. Know thyself: well-being and subjective experience. Cerebrum. 2018;2018. PMC6353121 30746034

[125] Damasio AR (1996). The somatic marker hypothesis and the possible functions of the prefrontal cortex. Philos Trans R Soc Lond B Biol Sci.

[126] Danziger K (1980). The history of introspection reconsidered. J Hist Behav Sci.

[127] Johansson P, Hall L, Sikström S, Olsson A (2005). Failure to detect mismatches between intention and outcome in a simple decision task. Science.

[128] Nisbett RE, Wilson TD (1977). Telling more than we can know: verbal reports on mental processes. Psychol Rev.

[129] Jerolmack C, Khan S (2014). Talk is cheap. Sociol Methods Res.

[130] Carruthers P. The opacity of mind: an integrative theory of self-knowledge. Oxford University Press; 2011.

[131] Robinson MD, Clore GL (2002). Belief and feeling: evidence for an accessibility model of emotional self-report. Psychol Bull.

[132] Walentynowicz M, Schneider S, Stone AA (2018). The effects of time frames on self-report. PLoS ONE.

[133] Chirimuuta M (2014). Psychophysical methods and the evasion of introspection. Philos Sci.

[134] Quigley KS, Lindquist KA, Barrett LF. Inducing and measuring emotion and affect: tips, tricks, and secrets. In: Reis HT, Judd CM, editors. Handbook of research methods in social and personality psychology. Cambridge University Press; 2014. p. 220–52.

[135] Borsboom D, Mellenbergh GJ (2004). The concept of validity. Psychol Rev.

[136] Cohen JA, Mannarino AP, Deblinger E. Treating trauma and traumatic grief in children and adolescents, 2nd ed. Guilford Publications; 2016.

[137] Schneider KJ, Krug OT. Existential-humanistic therapy. American Psychological Association Washington, DC; 2010.

[138] Hofmann SG, Gómez AF (2017). Mindfulness-based interventions for anxiety and depression. Psychiatric Clin.

[139] Zoellner LA, Foa EB (2016). Applying Research Domain Criteria (RDoC) to the study of fear and anxiety: a critical comment. Psychophysiology.

[140] Constantinou E, Purves KL, McGregor T, Lester KJ, Barry TJ, Treanor M (2021). Measuring fear: association among different measures of fear learning. J Behav Ther Exp Psychiatry.

[141] Nord CL, Barrett LF, Lindquist KA, Ma Y, Marwood L, Satpute AB (2021). Neural effects of antidepressant medication and psychological treatments: a quantitative synthesis across three meta-analyses. Br J Psychiatry.

[142] Taschereau-Dumouchel V, Kawato M, Lau H (2020). Multivoxel pattern analysis reveals dissociations between subjective fear and its physiological correlates. Mol Psychiatry.

[143] FeldmanHall O, Glimcher P, Baker AL, PROSPEC NYU Collaboration, Phelps EA. The functional roles of the amygdala and prefrontal cortex in processing uncertainty. J Cogn Neurosci. 2019;31:1742–54. 10.1162/jocn_a_01443 PMC7266503 31298634

[144] Hofmann SG, Curtiss JE, Hayes SC (2020). Beyond linear mediation: toward a dynamic network approach to study treatment processes. Clin Psychol Rev.

[145] LeDoux JE, Lau H (2020). A new vista in psychiatric treatment: using individualized functional connectivity to track symptoms. Proc Natl Acad Sci USA.

[146] Sylvester CM, Yu Q, Srivastava AB, Marek S, Zheng A, Alexopoulos D (2020). Individual-specific functional connectivity of the amygdala: a substrate for precision psychiatry. Proc Natl Acad Sci USA.

[147] Allen JG, Haccoun DM (1976). Sex differences in emotionality: a multidimensional approach. Hum Relat.

[148] Gard MG, Kring AM (2007). Sex differences in the time course of emotion. Emotion.

[149] Zhou F, Zhao W, Qi Z, Geng Y, Yao S, Kendrick KM (2021). A distributed fMRI-based signature for the subjective experience of fear. Nat Commun.

[150] Ekman P (1992). An argument for basic emotions. Cognition Emot.

[151] Scherer KR (2009). The dynamic architecture of emotion: evidence for the component process model. Cognition Emot.

[152] Barrett LF. How emotions are made: the secret life of the brain. Houghton Mifflin Harcourt; 2017.

[153] Mashour GA, Roelfsema P, Changeux J-P, Dehaene S (2020). Conscious processing and the global neuronal workspace hypothesis. Neuron.

[154] Apkarian AV (2019). Definitions of nociception, pain, and chronic pain with implications regarding science and society. Neurosci Lett.

[155] Krueger RF, Hobbs KA (2020). An overview of the DSM-5 alternative model of personality disorders. Psychopathology.

[156] Michel M, Lau H. On the dangers of conflating strong and weak versions of a theory of consciousness. Philos. Mind Sci. 2020;1.

[157] Dretske F. Naturalizing the mind. Cambridge, MA: Bradford; 1995.

[158] Lamme VAF (2010). How neuroscience will change our view on consciousness. Cogn Neurosci.

[159] Tye M. Consciousness, color, and content (representation and mind). MIT Press; 2000.

[160] Block N. Empirical science meets higher order views of consciousness: reply to Hakwan Lau and Richard Brown. Blockheads! Essays on Ned block’s philosophy of mind and consciousness. MIT Press Cambridge, MA; 2019. p. 199–213.

[161] Panksepp J (2012). What is an emotional feeling? Lessons about affective origins from cross-species neuroscience. Motiv Emot.

[162] Lau H, Rosenthal D (2011). Empirical support for higher-order theories of conscious awareness. Trends Cogn Sci.

[163] Brown R, Lau H, LeDoux JE (2019). Understanding the higher-order approach to consciousness. Trends Cogn Sci.

[164] Fleming SM, Daw ND (2017). Self-evaluation of decision-making: a general Bayesian framework for metacognitive computation. Psychol Rev.

